# Identifying atheroprotective fruits and vegetables by Mendelian Randomization analysis

**DOI:** 10.3389/fnut.2024.1426763

**Published:** 2024-10-14

**Authors:** Shenji Yang, Zhikang Cao, Huidong Liu, Zhipeng Li, Shaoping Nie, Mingyong Xie

**Affiliations:** ^1^State Key Laboratory of Food Science and Resources, China-Canada Joint Lab of Food Science and Technology (Nanchang), Key Laboratory of Bioactive Polysaccharides of Jiangxi Province, Nanchang University, Nanchang, China; ^2^Department of Medical Imaging, Ganzhou People's Hospital, The Affiliated Ganzhou Hospital of Nanchang University, Ganzhou, China

**Keywords:** fruits and vegetables, atherosclerosis, Mendelian Randomization, LDL-C and TG, C-reactive protein

## Abstract

**Background:**

Fruits and vegetables (FVs) are widely believed to mitigate the risk of atherosclerosis (AS). However, the causal relationships between specific FVs and AS risk factors remain unclear.

**Methods:**

This study performed two-sample Mendelian Randomization (MR) analysis to infer the causality of the intake of 28 kinds of FVs with AS, as well as its risk factors including blood low-density lipoprotein cholesterol (LDL-C), triglycerides (TG) and C-reactive protein (CRP). GWAS genetic data for these exposures and outcomes were extracted from the IEU open GWAS project. Heterogeneity was evaluated using both Inverse Variance Weighted (IVW) and MR-Egger methods. MR-Egger regression was specifically deployed to detect potential pleiotropy. Furthermore, a “leave-one-out” sensitivity analysis was conducted to determine the impact of each individual single nucleotide polymorphism (SNP) on the combined outcome.

**Results:**

The analysis confirms a causal relationship between total fruit consumption and reduced levels of LDL-C (OR = 0.911, *p* = 0.007) and CRP (OR = 0.868, *p* = 0.008). Similarly, total vegetable intake is also causally associated with a reduction in CRP levels (OR = 0.858, *p* = 0.018). Specifically, garlic intake exhibits the most significant causal relationship with reduced risk of AS (OR = 0.985, *p* = 0.036) and also causally associated with lower levels of LDL-C and TG. Berry (OR = 0.929, *p* = 0.010) and potato (OR = 0.957, *p* = 0.020) intake both display a significant causal negative association with TG levels, while peach/nectarine consumption is significantly associated with reduced CRP levels (OR = 0.913, *p* = 0.010).

**Conclusion:**

This is the first MR study that systemically examined the causality between commonly consumed FVs and AS. Our findings highlight the atheroprotective effects of various FVs, particularly garlic, on cardiovascular health and the importance of tailored nutritional recommendations to prevent AS.

## Introduction

Atherosclerosis (AS) is a pathological state characterized by the localized accumulation of cholesterol and other lipids primarily within the intimal layer of medium and large arteries (1). In 2019, it was estimated that cardiovascular diseases (CVD) were responsible for the deaths of ~17.9 million people, representing 32% of all global fatalities (2). The majority of these deaths stemmed from myocardial infarction and stroke, which are often consequences of AS (3). AS usually progresses insidiously over decades, and it might not produce noticeable symptoms until there is considerable narrowing of the arteries or an abrupt plaque rupture (4). Being a disease influenced by multiple factors, including genetic predisposition, lifestyle choices, and metabolic disturbances, AS presents challenges in devising a one-size-fits-all treatment strategy. Managing AS typically involves significant lifestyle modifications, particularly with respect to diet and physical activity (5). Diets rich in fruits and vegetables (FVs) have been linked to a lower risk of developing atherosclerotic conditions (6). Epidemiological research has consistently demonstrated an inverse association between such dietary patterns and subclinical markers of AS, including carotid intima-media thickness (IMT) and the presence of atherosclerotic plaques (7). For instance, Ellingsen et al. calculated the intake of total fruits and berries in elderly men using a food frequency questionnaire (FFQ) and measured their carotid IMT (8). They found that carotid IMT was significantly lower in the highest quartile of fruit and berry intake compared to the lowest quartile. This inverse association held even after adjustments for age, smoking, and other dietary components. Similarly, Blekkenhorst et al. quantified the total vegetable intake of older adult women with subclinical AS using FFQ and discovered that participants consuming ≥3 servings of vegetables had significantly 4.6–5.0% lower carotid IMT compared with those consuming <2 servings (7).

Fruits and vegetables are typically rich in dietary fiber and various phytochemicals, such as plant sterols and flavonoids. Dietary fiber can slow gastric emptying and the transit of food through the intestines, reducing the absorption of fats and cholesterol, which assists in lowering blood lipid levels (9). Furthermore, dietary fiber can regulate the composition of gut commensals, which plays a pivotal role in the regulation of metabolism and immune responses across multiple organs and tissues (10, 11). Plant sterols, which structurally resemble cholesterol, can compete with it for intestinal absorption (12), while flavonoids can enhance vascular function and exert anti-inflammatory effects (13, 14). Additionally, Vitamins C and E, along with β-carotene and various other antioxidants, are essential in mitigating oxidative stress, which is a significant contributing factor in the development of AS. Oxidative stress promotes the oxidation of low-density lipoprotein (LDL), a critical step that leads to inflammatory responses and the eventual buildup of atherosclerotic plaques within the arteries (15). Moreover, FVs are rich sources of potassium and magnesium, minerals that are essential for maintaining normal blood pressure, a significant risk factor for AS (16). In fact, specific fruits such as apples, bananas, berries, cherries, grapes, grapefruits, melons, and, plums (17–24), as well as vegetables like cabbage, carrots, celery, garlic, onions, and tomatoes (25–30) have been associated, to varying degrees, with a protective effect against AS. However, findings remain inconsistent, and a definitive causal link between the consumption of these FVs and AS has yet to be established. Furthermore, since each type of fruit and vegetable has a unique phytochemical profile, it is still unclear which are the most effective at reducing the risk of AS. The systematic evaluation and comparison of the causal relationships between the consumption of various specific FVs and the development of AS require further investigation. The impact of dietary components on health may become evident only over a prolonged timeframe, complicating the assessment of long-term effects. Therefore, conducting randomized double-blind clinical trials to explore these potential causal links poses significant challenges.

Mendelian Randomization (MR) is a statistical method that uses genetic variants as instrumental variables (IVs) to infer causal relationships between risk factors and health outcomes. This approach assumes that the genetic variants are associated with the exposure but not with any confounders, enabling estimates of direct causal effects. In the present study, we utilized a two-sample MR method to examine and compare the potential causal relationships between the consumption of various FVs and the risk of developing AS. We also assessed the impact of these dietary elements on AS risk factors, including blood lipid levels and chronic inflammation. The fruits analyzed in this study were apples, bananas, berries, cherries, grapes, grapefruits, mangoes, melons, oranges, peaches/nectarines, pears, pineapples, and plums. The vegetables included broccoli, cabbage/kale, carrots, cauliflower, celery, courgettes, cucumbers, garlic, green beans, lettuce, onions, potatoes, spinach, sweet peppers, and tomatoes. This study underlines the potential for more tailored dietary recommendations in the prevention and management of AS.

## Materials and methods

### Study design

**Figure 1:**
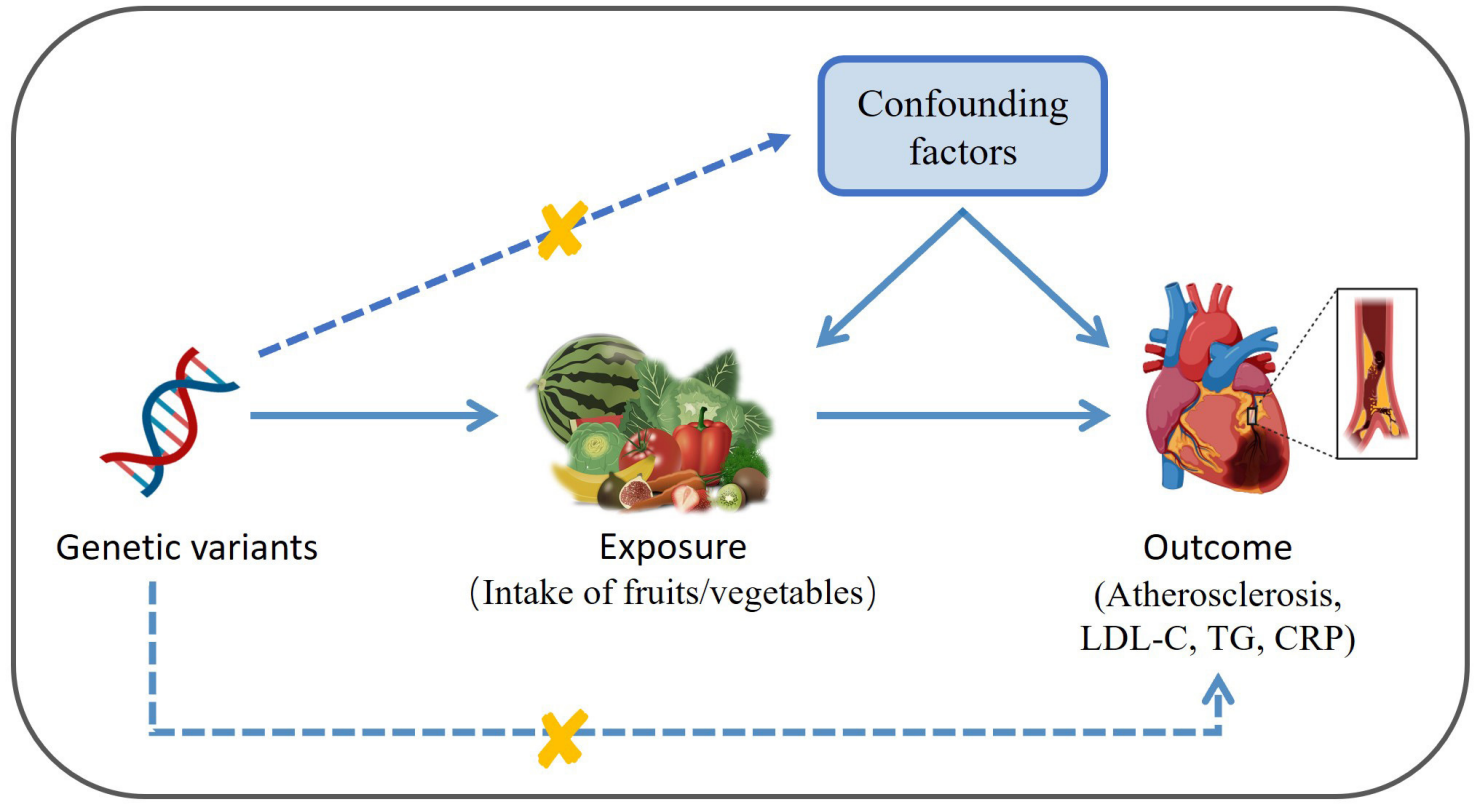
Diagram depicting the model of two-sample Mendelian Randomization analysis of the causal links between the intake of various fruits/vegetables and atherosclerosis as well as its risk factors (created with BioRender.com). LDL-C, low density lipoprotein cholesterol; TG, triglyceride; CRP, C-reactive protein.

As depicted in Figure 1, this study performed to two-sample MR analysis to infer the causality between the consumption of various fruits and vegetables (FVs) and AS, as well as its risk factors including the levels of LDL-C, TG and CRP. FV consumption was used as exposure and AS, LDL-C, TG, and CRP were used as outcome. MR analysis followed three core assumptions: genetic variation is strongly associated with exposure; the genetic variation is not associated with confounding factors; the genetic variation influence outcome only through the exposure.

### Data source

GWAS datasets for FV intake, AS, LDL-C, and C-reactive protein (CRP) were obtained from publicly available database, the IEU Open GWAS project (https://gwas.mrcieu.ac.uk/), with detailed information provided in Supplementary Table S1. Genetic datasets for the intake of various FVs were extracted from the IEU analysis of UK Biobank phenotypes. The datasets for the intake of various FVs included data from 64,949 participants, while those for total fruit and total vegetable intake involved 446,462 and 435,435 participants, respectively. In addition, genetic data for coronary atherosclerosis (ukb-d-I9_CORATHER) included 346,860 participants, comprising 14,334 cases and 332,526 controls. Genetic data for LDL-C (ieu-b-110), triglycerides (TG) (ieu-b-111), and CRP (ebi-a-GCST90029070) were collected from 440,546, 441,016, and 427,367 individuals, respectively. Participants included both males and females, all of whom were of European descent. The original genetic datasets for FV intake utilized ordered categorical phenotypes, while for LDL-C, TG, and CRP, continuous phenotypes were used. The dataset for AS employed binary phenotypes. For LDL-C and TG, age, sex, and genotype chip were used as covariates.

### Selection of instrumental variables

In order to identify SNPs that can act as IVs for the consumption of specific FVs, the following criteria were established: **Association with FV intake**: The SNP must show a statistically significant association with the intake of a specific FV (*p* < 5 × 10^−6^). For SNPs associated with the consumption of total fruits or vegetables, a more stringent significance level of *p* < 5 × 10^−7^ is required. **Association with outcome**: the SNP must also be associated with the outcome of interest (AS and risk factors) with a *p*-value > 10^−4^. This ensures that the SNP is not too strongly linked with the outcome, which could confound the causal inference. **Linkage disequilibrium (LD) minimization**: to ensure that identified SNPs are independent from one another, an LD threshold of *R*^2^ <0.01 is set within a 5,000-kilobase (kb) window. This minimizes the possibility that the SNP associations are due to LD rather than a direct relationship with FV intake. The 5 super-populations in the 1,000 genomes project were used as a reference panel. SNPs that are missing from the reference panel were excluded from the analysis. **Instrument strength**: the strength of the IVs is assessed using the *F*-statistic. An *F*-value of <10 is indicative of a weak instrument, which could introduce significant bias into the causal estimates. Such weak IVs should be excluded from the analysis to avoid misleading conclusions (31). **Confounding factors**: to mitigate the influence of potential confounders, we conducted a Phenome-wide association analysis using tools provided by the IEU Open GWAS project. This analysis involved searching for the effects of specific genetic variants across all publicly available datasets. Any SNPs that showed significant (*p* < 5 × 10^−6^) associations with potential confounders like meat, dairy, tobacco, and alcohol were excluded.

### MR analysis approach

For the performance of two-sample MR analyses, R software (version 4.3.1) along with the TwoSampleMR package (version 0.5.7) were utilized. The effects of selected IVs were harmonized to ensure the effect of a SNP on the exposure and the effect of that SNP on the outcome must each correspond to the same allele. The Inverse Variance Weighted (IVW) method was adopted as the main analytical approach, under the assumption that each SNP acts as a valid IV. Complementary methods, including MR Egger and the weighted median, were also implemented. The choice between fixed and mixed random effects models was dependent on the presence of heterogeneity; the mixed random effects model was adopted in the case of detected heterogeneity, while the fixed effect model was employed otherwise. The outcomes of these analyses were expressed as β (effect size) and odds ratios (OR) with corresponding 95% confidence intervals (CI), thereby quantifying the strength and precision of the inferred causal relationships. After the analysis, the multiple test results were also adjusted for false discovery rate (FDR) using the Benjamini-Hochberg (BH) procedure. Statistical significance was defined as a *p*-value that was lower than 0.05.

### Heterogeneity, pleiotropy, and sensitivity test

To ensure the reliability of our results, multiple tests were carried out to evaluate heterogeneity, pleiotropy, and sensitivity. Heterogeneity was evaluated using both Inverse Variance Weighted (IVW) and MR-Egger methods. To detect any potential horizontal pleiotropy, MR-Egger regression was specifically employed. If the intercept does not significantly differ from 0 (*p* > 0.05), it is considered that there is no sign of pleiotropy. Furthermore, a “leave-one-out” sensitivity analysis was conducted to determine the impact of each individual SNP on the combined outcome. This analysis entailed recalculating the effect estimates while systematically excluding one SNP at a time.

## Results

### Association between various FVs and AS

**Figure 2:**
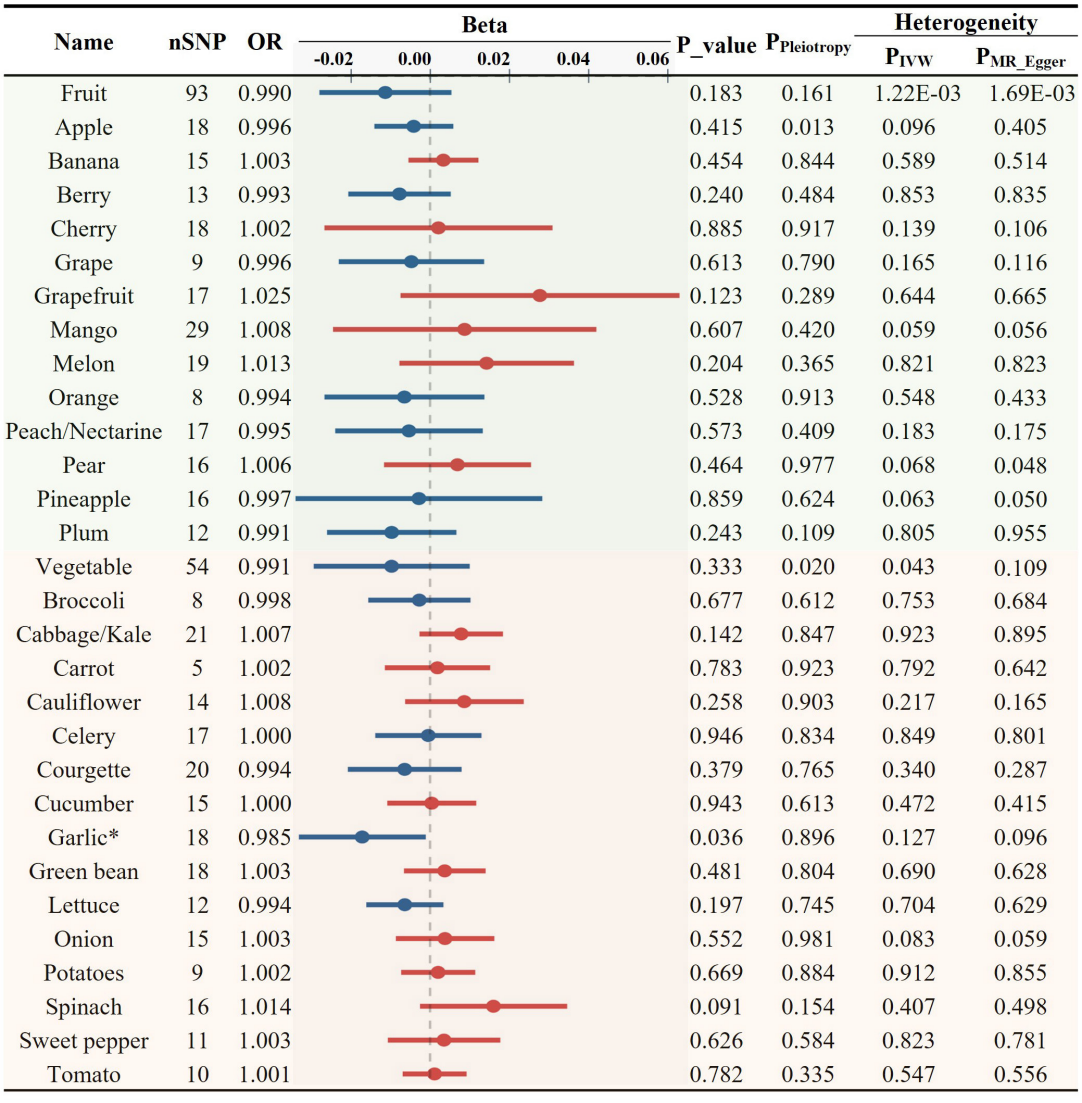
Causal relationships between fruit and vegetable consumption and atherosclerosis risk (*p < 0.05).

Initially, we investigated the potential causal relationship between the total consumption of fruits or vegetables and the risk of AS. No significant associations were observed for either category (fruit OR = 0.990, *p* = 0.183; vegetable OR = 0.991, *p* = 0.333, Figure 2). Subsequently, we examined the associations between specific FVs and AS. All the results of MR analyses for various FV on AS and its risk factors, including heterogeneity and pleiotropy tests are provided in Supplementary Table S2. After applying predetermined screening criteria, 745 SNPs were identified as IVs for analyzing the consumption of 28 different FVs. The *F*-statistics for each of these selected IVs exceeded the threshold of 10, indicating a minimal risk of weak instrument bias. Detailed information about these IVs is provided in Supplementary Table S3. When evaluating 13 distinct types of fruit, we found no significant association with AS (Figure 2; Supplementary Table S2). Among the 15 tested vegetable varieties, the intake of garlic showed the most significant causal association with a decreased risk of AS (OR = 0.985, *p* = 0.036, Figures 2, 6A; Supplementary Table S2). Despite previous reports suggesting an antiatherogenic effect of onions and tomatoes (30, 32), our analysis did not detect any significant association between their consumption and the risk of AS. A sensitivity test was conducted using the leave-one-out method, and it was found that no single IV significantly impacted the results (Supplementary Table S4).

### Association between various FVs and blood lipids

**Figure 3:**
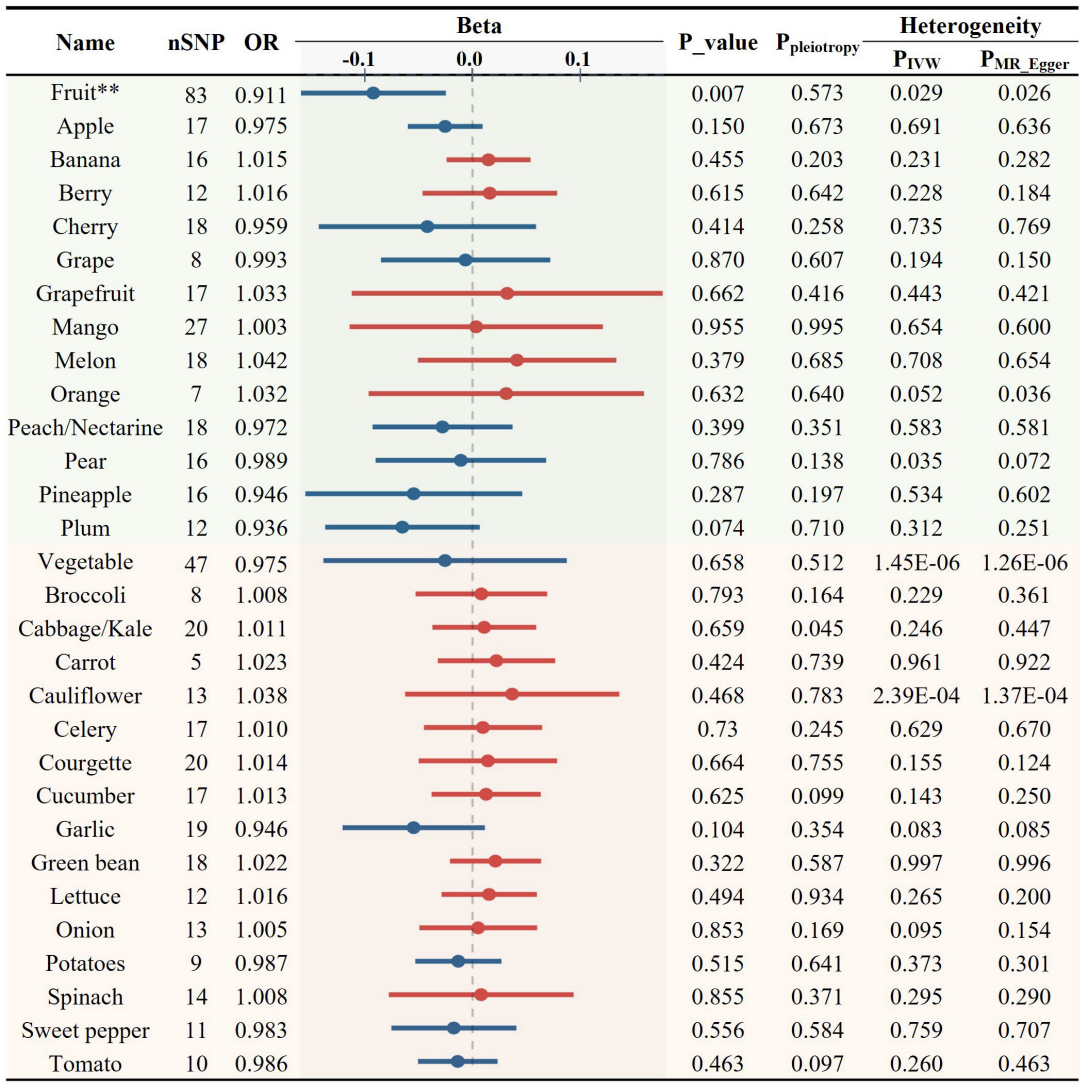
Causal relationships between fruit and vegetable consumption and the levels of circulating low-density lipoprotein cholesterol (**p < 0.01).

As elevated levels of LDL-C and TG are recognized risk factors for AS, we further examined the causal relationships between the total intake of fruits/vegetables and blood lipid profiles. Fruit consumption was significantly associated with reduced levels of LDL-C (OR = 0.911, *p* = 0.007, Figures 3, 6B) while no significant association was found with total vegetable consumption (Figure 3).

**Figure 4:**
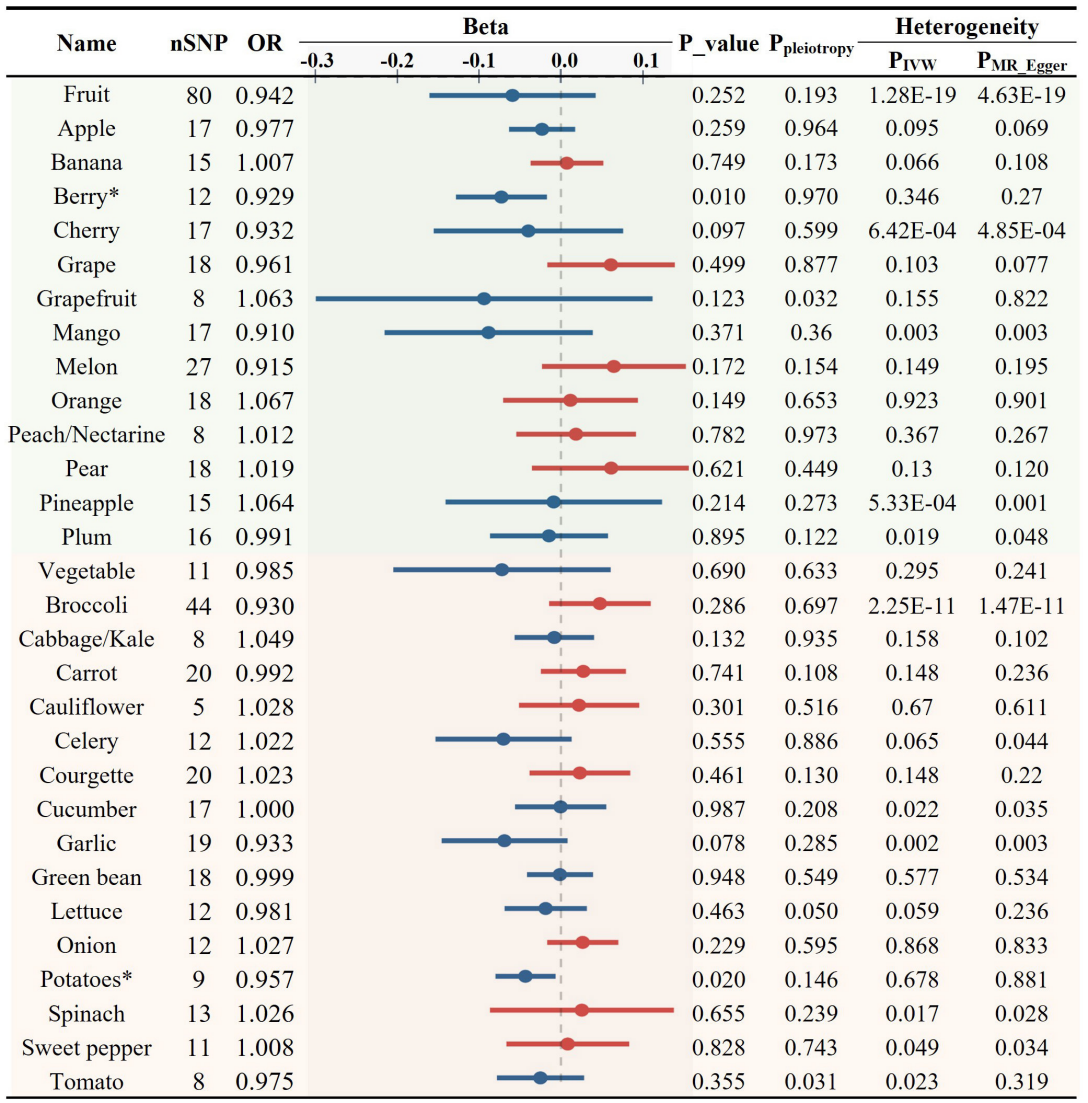
Causal relationships between fruit and vegetable consumption and the levels of blood triglyceride (*p < 0.05).

Next, we used 408 SNPs as IVs for LDL-C and 401 SNPs for TG to investigate the impact of 28 specific FVs on lipid levels (Supplementary Tables S5, S6). As shown in Figures 3, 4 and Supplementary Table S2, garlic intake displayed a negative association with the levels of LDL-C (OR = 0.946, *p* = 0.104) and TG (OR = 0.933, *p* = 0.078), which is consistent with its negative association with AS. Additionally, plum consumption had a marginally negative association with LDL-C (OR = 0.936, *p* = 0.074, Figure 3). Moreover, berry consumption was significantly and inversely associated with TG levels (OR = 0.929, *p* = 0.010, Figures 4, 6C). The intake of potatoes also showed a significant negative association with TG levels (OR = 0.957, *p* = 0.020, Figure 4). Sensitivity assessment revealed that the exclusion of any single IV did not substantially alter the findings (Supplementary Tables S7, S8). These outcomes suggest that certain FVs may have cholesterol and TG-lowering effects.

### Association between various FVs and inflammation

**Figure 5:**
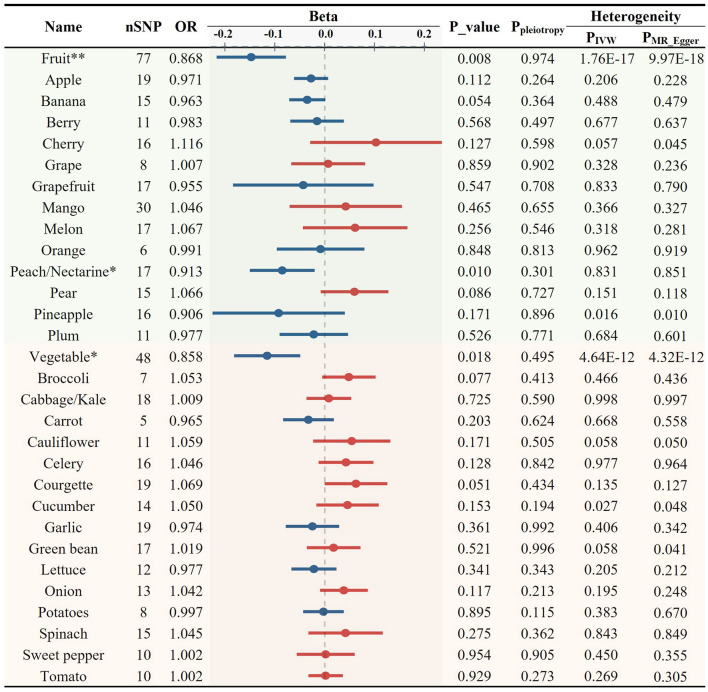
Causal relationships between fruit and vegetable consumption and the levels of C-reactive protein (*p < 0.05, **p < 0.01).

**Figure 6:**
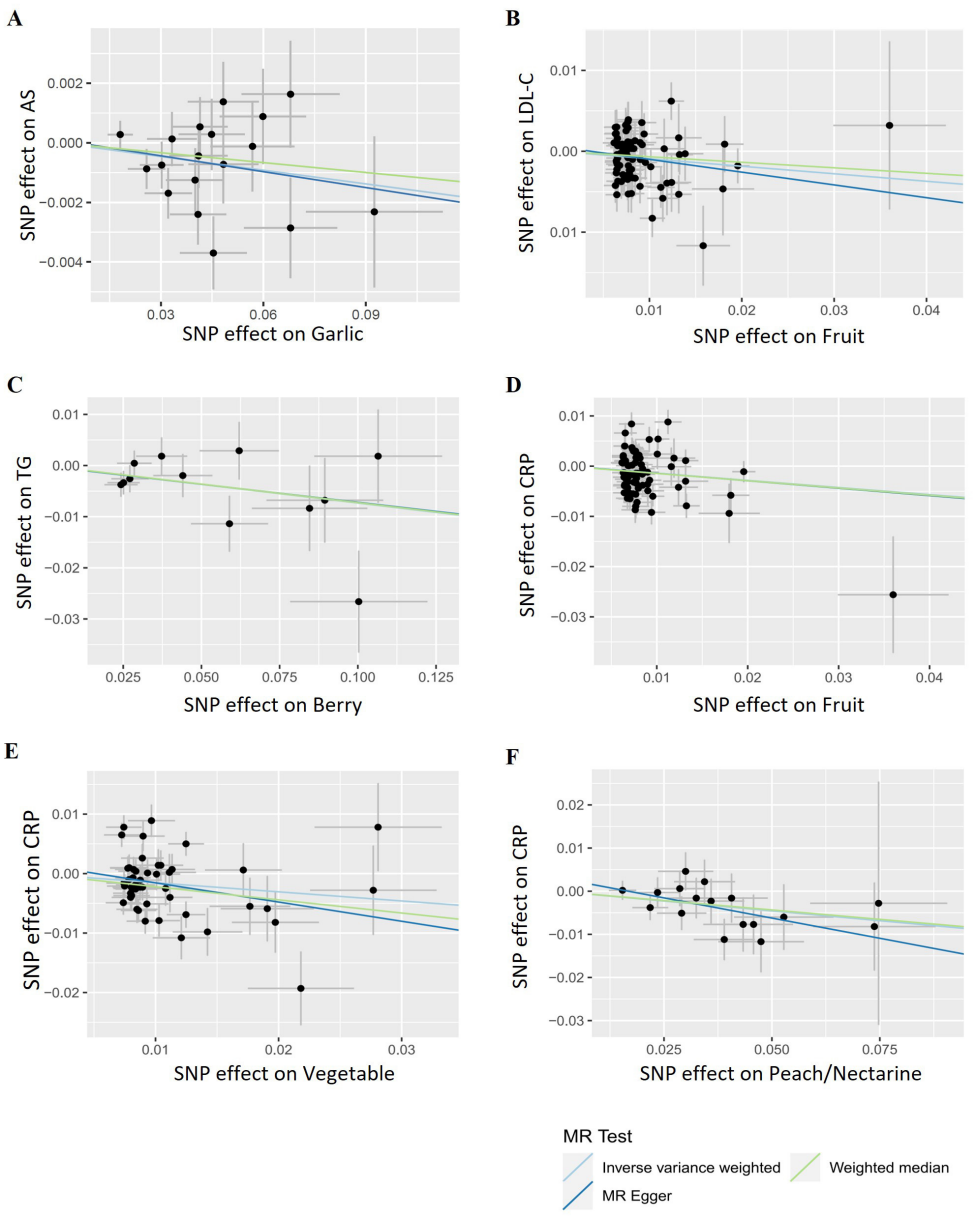
Representative scatter plots of the causal relationships between fruits/vegetables and atherosclerosis as well as its risk factors. **(A)** Garlic and atherosclerosis. **(B)** Fruit and LDL-C. **(C)** Berry and TG. **(D)** Fruit and C-reactive protein (CRP). **(E)** Vegetable and CRP. **(F)** Peach/nectarine and CRP.

Inflammation plays an important role in the development and progression of AS. CRP serves as a marker of chronic inflammation. High-sensitivity CRP test is particularly useful for the assessment of CVD risk. We found both consumption of total fruits (OR = 0.868, *p* = 0.008) and vegetables (OR = 0.858, *p* = 0.018) showed a significant negative causal association with CRP levels (Figures 5, 6D, E). Upon the application of predetermined screening criteria, a total of 392 SNPs with *F*-statistics > 10 were identified as IVs for the analysis of 28 FVs. Detailed information regarding these IVs is presented in Supplementary Table S9. Out of the 28 FVs, peach/nectarine consumption showed the most significant negatively causal association with the levels of CRP (OR = 0.913, *p* = 0.010, Figures 5, 6F; Supplementary Table S2). Consistent with its negative association with AS and the levels of LDL-C and TG, garlic also showed a trend to be negatively associated with CRP (OR = 0.974, *p* = 0.361, Figure 5). Sensitivity test demonstrated that omitting each IV in turn did not notably influence the overall results (Supplementary Table S10).

## Discussion

CVDs remain the leading cause of diet-associated mortality and morbidity worldwide (33). An insufficient intake of fruits and vegetables is a major contributor to cardiometabolic deaths (34). Fruits and vegetables are abundant sources of dietary fiber, essential micronutrients, and a range of bioactive compounds, including vitamins, minerals, polyphenols, carotenoids, and organosulfur compounds (35). These elements may play a critical role in preventing the development of CVD and in modulating intermediary risk factors such as blood lipid levels, blood pressure, and body mass (36). Chronic inflammation is closely linked to the development of AS. LDL-C in the bloodstream can become oxidized through inflammatory processes, leading to its adherence to arterial walls and increasing its atherogenic potential. Monocytes that enter the arterial wall can transform into macrophages, ingest oxidized LDL, and become foam cells, which are a hallmark of early atherosclerotic plaques (37).

In this work, we utilized two-sample MR analysis approach to investigate the causality between the intake of various FVs and AS, as well as its risk factors, including blood lipids, and chronic inflammation. Although not statistically significant, the overall intake of fruits and vegetables appeared to exhibit negative causal associations with the risk of AS. Among the 13 types of fruit and 15 types of vegetables studied, garlic exhibited the most significant negative causal association with AS. Moreover, our results also suggest a marginally significant inverse causal relationship between garlic intake and levels of circulating LDL-C and TG in humans.

Prior research has shown that garlic compounds can reduce intracellular oxidative stress and inhibit NF-κB activation (38, 39). A clinical trial reported that long-term supplementation with aged garlic extract improved peripheral tissue perfusion in patients with AS (40). Moreover, a meta-analysis suggested that garlic supplementation may lower the levels of serum TC and LDL-C (41). According to literature review, garlic is one of the vegetables with the strongest protective effects against AS. Consistently, garlic showed the strongest atheroprotective effect among the FVs examined in this study. It is always valuable to validate biological findings using different approaches.

Mechanisms have been reported on how garlic may improve AS. Garlic contains bioactive compounds such as allicin, diallyl disulfide (DADS), and S-allyl cysteine (SAC), etc. (42). Allicin can inhibit the activation of JNK and p38 MAPK and decrease SR-A and CD36 expression, thereby preventing macrophage foam formation (43). It also activates PPARγ/LXRα signaling in THP-1 macrophage-derived foam cells, thereby reducing lipid accumulation. Additionally, allicin promotes cholesterol efflux through upregulation of ATP Binding Cassette Subfamily A Member 1 (ABCA1) (44). It has also been suggested that alliin could inhibit platelet aggregation and prevent the progression of AS (42). DADS inhibits hepatic lipid synthesis by downregulating SREBP-1c expression and promote lipolysis and fatty acid oxidative metabolism by upregulating the PPARα in hepatocytes. SAC can also inhibit SREBP-1 mediated lipogenesis by activating AMPK through calcium/calmodulin-dependent kinase and silent information regulator T1 (45). In addition, the molecular mechanisms involved in the antioxidant, anti-inflammatory, antithrombotic, and endothelium-protective effects of garlic compounds have also been demonstrated (42).

While some clinical studies suggest that tomato supplementation can lower serum TC, TG, and blood pressure, there are still discrepancies in the results (30). Additionally, these human trials typically involve small sample sizes and do not accurately model AS. Smith et al. employed ApoE^−/−^ mouse model to investigate the anti-atherosclerotic properties of tomatoes and observed no significant effects (46). Although animal studies indicate that onion extract might decrease arterial lesions and lipid levels and potentially improve AS by affecting various pathways (47), clinical trials to confirm these effects are lacking (46). In our study, neither tomatoes nor onions demonstrated a clear relationship with AS, blood lipid levels, or CRP levels. They contain functional compounds such as lycopene and quercetin, which are theoretically atheroprotective (48). The amount consumed may be a critical factor in determining their effectiveness.

Berries are rich in anthocyanins, such as cyanidin, pelargonidin, delphinidin, peonidin, malvidin, and petunidin, which are known for their antioxidative activity (49). They have been extensively reviewed for their protective effects against CVD. A meta-analysis involving 41 studies and 2,788 participants showed that anthocyanin supplementation significantly reduced TG and LDL-C levels (50). Consistent with these findings, our study detected a significant negative causal association between berry consumption and TG levels. Animal studies have demonstrated mechanistic insights into the role of berries in lipid metabolism. It has been revealed that polyphenolic flavonoids and phenolic acids found in berries enhance the activity of paraoxonase, which is linked to the antioxidant properties of HDL cholesterol, and also boost the liver's production of apolipoprotein A-I (51). Commonly consumed berries, such as blueberries and strawberries, have been demonstrated to suppress fatty acid synthesis, reduce aortic lesions, and alleviate both inflammation and oxidative damage. The polyphenol content and profile are important factors in determining the hypolipidemic effect of berries (52).

Potatoes, being starchy vegetables, have been the subject of conflicting reports regarding their impact on TG levels. Although a cohort study reported only weak associations with high TG levels following boiled potato consumption (53), another study indicated that a steamed potato-enriched diet significantly decreased cholesterol and TG levels in rats, compared to a wheat starch-enriched diet (54). Our findings support a causal link between the consumption of potatoes and lowered TG levels.

A diet rich in fruits and vegetables is beneficial for reducing inflammation, which is attributed to the high content of antioxidants and biologically active substances present in these foods. Our analysis revealed that the consumption of both fruits and vegetables can potentially reduce inflammation. Peaches, in particular, contain a broad spectrum of phytochemicals, including phenolic compounds and carotenoids, as well as vitamins, volatile aroma compounds, and organic acids (55). Research has shown that both fresh and preserved peach supplements can reduce oxidative stress, inflammation, and tissue damage caused by CCl4. They inhibit the activation of inflammatory mediators such as TNF-α, IL-1β, RAGE, and NF-κB (56). Another study demonstrated that consumption of polyphenol-rich peach and plum juice may prevent risk factors for obesity-related metabolic disorders and cardiovascular disease in Zucker rats (57). Consistent with these studies, our research found a significant causal association between peach/nectarine consumption and reduced CRP levels in humans.

We primarily used two-sample MR analysis to explore the direct relationships between individual FV intakes and AS risks. This approach could not capture the potential interactions among these dietary components. Therefore, we further performed multivariable MR (MVMR) analysis on the significant FVs to assess the independent effect of each FV, considering the influence of other FVs on the outcomes. As shown in Supplementary Table S11, garlic intake still shows a negative causal association with AS (OR = 0.985, *p* = 0.009) and TG (OR = 0.940, *p* = 0.049). Berry intake continues to be negatively associated with TG (OR = 0.934, *p* = 0.024). Similarly, peach intake remains negatively associated with CRP levels (OR = 0.900, *p* = 0.012). Overall, the effect size and significance levels remained similar to those observed in previous analyses.

Some limitations in this study should also be noted. Firstly, it is challenging to fully consider the complex interactions given the complexity of dietary compositions. Future studies may benefit from exploring these interactions more comprehensively using advanced statistical models and larger datasets. Second, population stratification can impact the results of MR analyses, as it may introduce bias where the association between genetic variants and environmental factors is mistakenly interpreted as causal relationships. Additionally, Heterogeneity was detected in some MR tests in this study. We adopted a random effects model to address this heterogeneity, which accounts for the potential differences in effect sizes among the various genetic instrumental variables. However, when interpreting these results, it is still necessary to carefully consider the potential impacts of heterogeneity. Our sample primarily consists of individuals of European descent, which may limit the generalizability of our findings. Future studies are needed to validate the results in different populations. Furthermore, intake amount and intake forms are key factors affecting the health benefits of FVs. However, due to the lack of relevant genetic datasets, this study could not provide a detailed relationship between these factors and the protective benefits. Therefore, the causal relationships revealed in this study and their underlying mechanisms require further in-depth investigation.

## Conclusion

This is the first study that systemically assesses and compares the atheroprotective effects of a diverse range of FVs. The findings indicate that, under MR assumptions, particular FVs may confer a protective effect against AS and its risk factors. Among the 28 FVs evaluated, garlic emerged as the one with the most pronounced negative causal relationship with the condition. Additionally, the consumption of berries and potatoes was notably associated with reduced TG levels. Regarding inflammation, a general intake of fruits and vegetables was linked with potential anti-inflammatory effects, with the consumption of peaches/nectarines specifically showing a significant and causal association with decreased CRP levels. Although the deeper mechanisms behind these causal relationships require further investigation, these discoveries shed light on the nuanced contributions of FVs to cardiovascular health and underscore the significance of consuming certain types of produce for their health advantages.

## Data Availability

The original contributions presented in the study are included in the article/Supplementary material, further inquiries can be directed to the corresponding author.
