# Supplementary material for: Identifying atheroprotective fruits and vegetables by Mendelian Randomization analysis
Source: Front Nutr. 2024 Oct 14;11:1426763. doi: 10.3389/fnut.2024.1426763 (PMC11513280; doi:10.3389/fnut.2024.1426763)
Supplement: Supplementary file 2 [file Table_2.docx]

**STROBE-MR checklist of recommended items to address in reports of Mendelian randomization studies**^1^ ^2^

| **Item No.** | **Section** | **Checklist item** | **Page No.** | **Relevant text from manuscript** |
| --- | --- | --- | --- | --- |
| 1 | **TITLE and ABSTRACT** | Indicate Mendelian randomization (MR) as the study’s design in the title and/or the abstract if that is a main purpose of the study |  | Identifying Atheroprotective Fruits and Vegetables by Mendelian RandomizationAnalysis |
|  | **INTRODUCTION** |  |  |  |
| 2 | **Background** | Explain the scientific background and rationale for the reported study. What is the exposure? Is a potential causal relationship between exposure and outcome plausible? Justify why MR is a helpful method to address the study question |  | Specific fruits and vegetables have been associated, to varying degrees, with a protective effect against AS. However, conflicts in the findings persist, and the causality between the consumption of these FVs and AS have not been conclusively established. Furthermore, it remains unclear which of these commonly consumed FVs are most effective in comparison to others. The systematic evaluation and comparison of the causal relationships between the consumption of various specific FVs and the development of AS require further investigation. The impact of dietary components on health may become evident only over a prolonged timeframe, complicating the assessment of long-term effects. Therefore, conducting randomized double-blind clinical trials to explore these potential causal links poses significant challenges.  In the present study, we utilized a two-sample MR method to examine and compare the potential causal relationships between the consumption of various FVs and the risk of developing AS. We also assessed the impact of these dietary elements on AS risk factors, including blood lipid levels and chronic inflammation. |
| 3 | **Objectives** | State specific objectives clearly, including pre-specified causal hypotheses (if any). State that MR is a method that, under specific assumptions, intends to estimate causal effects |  | Mendelian Randomization (MR) is a statistical method that uses genetic variants as instrumental variables (IVs) to infer causal relationships between risk factors and health outcomes. This approach assumes that the genetic variants are associated with the exposure but not with any confounders, enabling estimates of direct causal effects. In the present study, we utilized a two-sample MR method to examine and compare the potential causal relationships between the consumption of various FVs and the risk of developing AS. We also assessed the impact of these dietary elements on AS risk factors, including blood lipid levels and chronic inflammation |
|  | **METHODS** |  |  |  |
| 4 | **Study design and data sources** | Present key elements of the study design early in the article. Consider including a table listing sources of data for all phases of the study. For each data source contributing to the analysis, describe the following: |  | Study design  Data source |
|  | a) | Setting: Describe the study design and the underlying population, if possible. Describe the setting, locations, and relevant dates, including periods of recruitment, exposure, follow-up, and data collection, when available. |  | As depicted in Fig. 1, this study performed to two-sample MR analysis to infer the causality between the consumption of various fruits and vegetables (FVs) and AS, as well as its risk factors including the levels of LDL-C, TG and CRP. GWAS datasets for FV intake, AS, LDL-C, and C-reactive protein (CRP) were obtained from the IEU Open GWAS project, with detailed dataset information provided in Table S1. |
|  | b) | Participants: Give the eligibility criteria, and the sources and methods of selection of participants. Report the sample size, and whether any power or sample size calculations were carried out prior to the main analysis |  | The data were extracted from public available database, IEU Open GWAS project, entailed dataset information provided in Table S1 |
|  | c) | Describe measurement, quality control and selection of genetic variants |  | ① **Association with FV Intake**: The SNP must show a statistically significant association with the intake of a specific FV. This is determined by a p-value lower than 5×10-6. For SNPs associated with the consumption of total fruits or total vegetables, a more stringent p-value threshold of less than 5×10-7 is required, reflecting an even lower probability of the association occurring by chance. ② **Association with Outcome**: The SNP must also be associated with the outcome of interest (AS and risk factors) with a p-value greater than 10-4. This ensures that the SNP is not too strongly linked with the outcome, which could confound the causal inference. ③ **Linkage Disequilibrium (LD) Minimization**: To ensure that identified SNPs are independent from one another, an LD threshold of R2<0.01 is set within a 5000-kilobase (kb) window. This minimizes the possibility that the SNP associations are due to LD rather than a direct relationship with FV intake. The 5 super-populations in the 1000 genomes project were used as a reference panel. ④ **Instrument Strength**: the strength of the IVs is assessed using the F-statistic. An F-value of less than 10 is indicative of a weak instrument, which could introduce significant bias into the causal estimates. Such weak IVs should be excluded from the analysis to avoid misleading conclusions(30) |
|  | d) | For each exposure, outcome, and other relevant variables, describe methods of assessment and diagnostic criteria for diseases |  | GWAS datasets for FV intake, AS, LDL-C, and C-reactive protein (CRP) were obtained from the IEU Open GWAS project (https://gwas.mrcieu.ac.uk/), with detailed dataset information provided in Table S1. |
|  | e) | Provide details of ethics committee approval and participant informed consent, if relevant |  | NA |
| 5 | **Assumptions** | Explicitly state the three core IV assumptions for the main analysis (relevance, independence and exclusion restriction) as well assumptions for any additional or sensitivity analysis |  | MR analysis followed three core assumptions: ① genetic variation is strongly associated with exposure; ② the genetic variation is not associated with confounding factors; ③ the genetic variation influence outcome only through the exposure. |
| 6 | **Statistical methods: main analysis** | Describe statistical methods and statistics used |  | MR analysis approach |
|  | a) | Describe how quantitative variables were handled in the analyses (i.e., scale, units, model) |  | The original genetic datasets for FV intake utilized ordered categorical phenotypes, while for LDL-C, TG, and CRP, continuous phenotypes were used. The dataset for AS employed binary phenotypes. |
|  | b) | Describe how genetic variants were handled in the analyses and, if applicable, how their weights were selected |  | Linkage Disequilibrium (LD) Minimization, The effects of selected IVs were harmonized (action=1) to ensure the effect of a SNP on the exposure and the effect of that SNP on the outcome must each correspond to the same allele. |
|  | c) | Describe the MR estimator (e.g. two-stage least squares, Wald ratio) and related statistics. Detail the included covariates and, in case of two-sample MR, whether the same covariate set was used for adjustment in the two samples |  | age, sex, and genotype chip were used as covariates. |
|  | d) | Explain how missing data were addressed |  | Linkage Disequilibrium (LD) Minimization, SNPs that are missing the reference panel were exclude from the analysis |
|  | e) | If applicable, indicate how multiple testing was addressed |  | After the analysis, the multiple test results were also adjusted for false discovery rate (FDR) using the Benjamini-Hochberg (BH) procedure. |
| 7 | **Assessment of assumptions** | Describe any methods or prior knowledge used to assess the assumptions or justify their validity |  | MR analysis approach. the Inverse Variance Weighted (IVW) method was designated as the main analytical approach, under the assumption that each SNP acts as a valid IV. Complementary methods, including MR Egger and the weighted median, were also implemented. |
| 8 | **Sensitivity analyses and additional analyses** | Describe any sensitivity analyses or additional analyses performed (e.g. comparison of effect estimates from different approaches, independent replication, bias analytic techniques, validation of instruments, simulations) |  | the strength of the IVs is assessed using the F-statistic  Heterogeneity, pleiotropy, and sensitivity test |
| 9 | **Software and pre-registration** |  |  |  |
|  | a) | Name statistical software and package(s), including version and settings used |  | For the performance of two-sample MR analyses, R software (version 4.3.1) along with the TwoSampleMR package (version 0.5.7) were utilized. |
|  | b) | State whether the study protocol and details were pre-registered (as well as when and where) |  | The study protocol was not pre-registered |
|  | **RESULTS** |  |  |  |
| 10 | **Descriptive data** |  |  |  |
|  | a) | Report the numbers of individuals at each stage of included studies and reasons for exclusion. Consider use of a flow diagram |  | Data source, Table S1 |
|  | b) | Report summary statistics for phenotypic exposure(s), outcome(s), and other relevant variables (e.g. means, SDs, proportions) |  | Table S1-2, S4, S6, S8 |
|  | c) | If the data sources include meta-analyses of previous studies, provide the assessments of heterogeneity across these studies |  | NA |
|  | d) | For two-sample MR:  i.  Provide justification of the similarity of the genetic variant-exposure associations between the exposure and outcome samples  ii.  Provide information on the number of individuals who overlap between the exposure and outcome studies |  | Exposure and outcome samples are not overlapping, as indicated in the “Data source” section |
| 11 | **Main results** |  |  |  |
|  | a) | Report the associations between genetic variant and exposure, and between genetic variant and outcome, preferably on an interpretable scale |  | This information is detailed in Table S2, S4, S5, S8 |
|  | b) | Report MR estimates of the relationship between exposure and outcome, and the measures of uncertainty from the MR analysis, on an interpretable scale, such as odds ratio or relative risk per SD difference |  | “Results” section, Fig2-5 showed odds ratio and forest plots. |
|  | c) | If relevant, consider translating estimates of relative risk into absolute risk for a meaningful time period |  | NA |
|  | d) | Consider plots to visualize results (e.g. forest plot, scatterplot of associations between genetic variants and outcome versus between genetic variants and exposure) |  | Representative scatterplots are provided in Fig. 6 |
| 12 | **Assessment of assumptions** |  |  |  |
|  | a) | Report the assessment of the validity of the assumptions |  | To ensure the reliability of our results, multiple tests were carried out to evaluate heterogeneity, pleiotropy, and sensitivity. Results have been shown in the “Results section” and supplementary tables. |
|  | b) | Report any additional statistics (e.g., assessments of heterogeneity across genetic variants, such as *I^2^*, Q statistic or E-value) |  |  |
| 13 | **Sensitivity analyses and additional analyses** |  |  |  |
|  | a) | Report any sensitivity analyses to assess the robustness of the main results to violations of the assumptions |  | leave-one-out" sensitivity analysis was conducted to determine the impact of each individual SNP on the combined outcome. |
|  | b) | Report results from other sensitivity analyses or additional analyses |  | Table S3,6,7,9 |
|  | c) | Report any assessment of direction of causal relationship (e.g., bidirectional MR) |  | NA |
|  | d) | When relevant, report and compare with estimates from non-MR analyses |  | Shown in “discussion” section |
|  | e) | Consider additional plots to visualize results (e.g., leave-one-out analyses) |  | Leave-one-out analyses results are shown Table S3,6,7,9 |
|  | **DISCUSSION** |  |  |  |
| 14 | **Key results** | Summarize key results with reference to study objectives |  | Conclusion, The findings indicate that particular FVs may confer a protective effect against AS and its risk factors. Among the 28 FVs evaluated, garlic emerged as the one with the most pronounced negative causal relationship with the condition… |
| 15 | **Limitations** | Discuss limitations of the study, taking into account the validity of the IV assumptions, other sources of potential bias, and imprecision. Discuss both direction and magnitude of any potential bias and any efforts to address them |  | Some limitations of this study should also be noted… |
| 16 | **Interpretation** |  |  |  |
|  | a) | Meaning: Give a cautious overall interpretation of results in the context of their limitations and in comparison with other studies |  | Although the deeper mechanisms behind these causal relationships require further investigation, these discoveries shed light on the nuanced contributions of FVs to cardiovascular health and underscore the significance of consuming certain types of produce for their health advantages… |
|  | b) | Mechanism: Discuss underlying biological mechanisms that could drive a potential causal relationship between the investigated exposure and the outcome, and whether the gene-environment equivalence assumption is reasonable. Use causal language carefully, clarifying that IV estimates may provide causal effects only under certain assumptions |  | Prior research has shown that garlic compounds can reduce intracellular oxidative stress and inhibit NF-κB activation…  The findings indicate that, under MR assumptions, particular FVs may confer a protective effect against AS and its risk factors… |
|  | c) | Clinical relevance: Discuss whether the results have clinical or public policy relevance, and to what extent they inform effect sizes of possible interventions |  | these discoveries shed light on the nuanced contributions of FVs to cardiovascular health and underscore the significance of consuming certain types of produce for their health advantages. |
| 17 | **Generalizability** | Discuss the generalizability of the study results (a) to other populations, (b) across other exposure periods/timings, and (c) across other levels of exposure |  | The results are based on the analysis of European populations, and caution is needed when generalizing to other populations…  Furthermore, intake amount and intake forms are crucial for the significant efficacy of FVs; however, due to the lack of relevant database, this study could not provide a detailed relationship between these factors and the protective benefits… |
|  | **OTHER INFORMATION** |  |  |  |
| 18 | **Funding** | Describe sources of funding and the role of funders in the present study and, if applicable, sources of funding for the databases and original study or studies on which the present study is based |  | Funding |
| 19 | **Data and data sharing** | Provide the data used to perform all analyses or report where and how the data can be accessed, and reference these sources in the article. Provide the statistical code needed to reproduce the results in the article, or report whether the code is publicly accessible and if so, where |  | “data source” section. Code is based on the Github TwoSampleMR instruction. |
| 20 | **Conflicts of Interest** | All authors should declare all potential conflicts of interest |  | The authors declare that the research was conducted in the absence of any commercial or financial relationships that could be construed as a potential conflict of interest. |

This checklist is copyrighted by the Equator Network under the Creative Commons Attribution 3.0 Unported (CC BY 3.0) license.

1. Skrivankova VW, Richmond RC, Woolf BAR, Yarmolinsky J, Davies NM, Swanson SA, et al. Strengthening the Reporting of Observational Studies in Epidemiology using Mendelian Randomization (STROBE-MR) Statement. JAMA. 2021;under review.

2. Skrivankova VW, Richmond RC, Woolf BAR, Davies NM, Swanson SA, VanderWeele TJ, et al. Strengthening the Reporting of Observational Studies in Epidemiology using Mendelian Randomisation (STROBE-MR): Explanation and Elaboration. BMJ. 2021;375:n2233.
